# The gut-brain axis as a therapeutic target in alcohol use disorder: dietary fiber, intestinal barrier integrity, and neuroinflammatory mechanisms underlying depression

**DOI:** 10.3389/fnut.2026.1927800

**Published:** 2026-09-01

**Authors:** Diliana Pérez-Reytor, Katherine García, Ítalo M. Urrutia, Nicolás Plaza, Eduardo Karahanian

**Affiliations:** 1 Institute of Biomedical Sciences, Faculty of Health Sciences, Universidad Autónoma de Chile, Santiago, Chile; 2 Research Center for the Development of Novel Therapeutic Alternatives for Alcohol Use Disorders, Universidad Autónoma de Chile, Santiago, Chile

**Keywords:** alcohol use disorder, butyrate, depression, dietary fiber, gut-brain axis, intestinal barrier, neuroinflammation, relapse

## Abstract

Alcohol use disorder is frequently accompanied by major depressive disorder, yet the biological mechanisms linking both conditions remain incompletely understood. Increasing evidence suggests that chronic alcohol consumption disrupts the gut-brain axis by damaging intestinal tight junctions and inducing gut dysbiosis characterized by depletion of short-chain fatty acid-producing bacteria. The resulting impairment of intestinal barrier integrity may facilitate the translocation of bacterial lipopolysaccharide, promoting peripheral and central neuroinflammation through Toll-like receptor 4-dependent pathways. These neuroinflammatory alterations may persist during abstinence and contribute to glutamatergic, dopaminergic, and serotonergic dysregulation associated with depressive symptoms and relapse vulnerability. This Perspective proposes that intestinal barrier dysfunction represents an actionable upstream mechanism linking alcohol-induced gut dysbiosis with neuroinflammation, depression, and relapse vulnerability, and highlights dietary fiber supplementation as a physiologically grounded, accessible nutritional strategy to enhance endogenous butyrate production, improve intestinal barrier integrity, and attenuate neuroinflammatory signaling. Available preclinical and clinical evidence is critically evaluated, and key priorities for future mechanistic and clinical studies are outlined.

## 1 Introduction

Alcohol use disorder (AUD) affects hundreds of millions of people worldwide and represents a leading contributor to global disability-adjusted life years (1). Among its most clinically challenging features is the high co-occurrence with major depressive disorder (MDD): more than one-third of individuals with AUD meet diagnostic criteria for MDD at some point during their illness trajectory (2). This comorbidity is associated with greater severity of depression, accelerated relapse to drinking, increased suicidal risk, and a significantly reduced response to conventional antidepressant treatment (3)—creating a vicious cycle in which each condition amplifies the other.

Despite decades of neuroscience research, the mechanistic link between AUD and MDD remains incompletely elucidated. Accumulating evidence supports the notion that alcohol-induced intestinal barrier dysfunction, gut dysbiosis, altered microbial metabolite production, and neuroimmune signaling constitute interconnected components of the gut-brain axis in AUD (4–6). Converging evidence now suggests that alcohol-induced disruption of the gut-brain axis promotes neuroinflammation, which may represent a shared pathophysiological mechanism linking alcohol use disorder and major depressive disorder (7, 8). Chronic alcohol consumption may damage the intestinal epithelial barrier, contribute to a dysbiotic shift in the gut microbiota that depletes butyrate-producing taxa, and enable bacterial lipopolysaccharide (LPS) to translocate into the systemic circulation. LPS activates Toll-like receptor 4 (TLR4)-mediated inflammatory cascades in the periphery and, subsequently, in the brain, leading to sustained neuroinflammation that disrupts glutamatergic, dopaminergic, and serotonergic homeostasis (8, 9). Critically, this neuroinflammatory state is not self-limiting upon alcohol cessation; it persists well into abstinence, maintaining the neurobiological conditions for depression and compulsive alcohol seeking (9, 10).

Within this framework, the depletion of short-chain fatty acids (SCFAs)—particularly butyrate, the principal colonocyte energy substrate and anti-inflammatory microbial metabolite emerges as a pivotal event linking dysbiosis to barrier dysfunction and downstream neuroinflammation (11–13). Unlike previous reviews that have examined gut dysbiosis, neuroinflammation, or SCFAs independently, this perspective integrates these mechanisms into a unified model linking alcohol-induced intestinal barrier dysfunction to neuroinflammation-driven depression and relapse vulnerability. Furthermore, we propose dietary fiber as an upstream therapeutic strategy that can restore endogenous butyrate production and interrupt this pathological cascade.

## 2 Defining depression in the context of AUD

Throughout this Perspective, the term depression refers to the depressive symptom burden commonly reported in individuals with AUD—including low mood, anhedonia, irritability, and craving-associated affective distress—as assessed using validated symptom scales in the cited clinical studies (14, 15), rather than to a DSM-5 diagnosis of major depressive disorder established by a structured clinical interview. We adopt this symptom-based framework because it more accurately reflects the clinical outcomes reported in the mechanistic and translational studies discussed throughout this article.

We also acknowledge that the neuroinflammatory and neurotransmitter alterations discussed here—including glutamatergic, dopaminergic, serotonergic, and BDNF-dependent mechanisms—are not specific to depression and likely contribute to other neuropsychiatric manifestations frequently associated with AUD, including anxiety, anhedonia, and craving (6). We focus on depressive symptoms because they are consistently associated with poorer treatment response and increased relapse risk in AUD (3), making them a clinically prioritized endpoint within this broader neurobiological framework. This Perspective does not propose that depression is mechanistically distinct from these related manifestations; rather, it uses depressive symptomatology as a clinically meaningful framework for discussing the therapeutic implications of targeting the gut-brain axis.

## 3 Alcohol-induced intestinal barrier disruption: a persistent biological injury

The intestinal epithelial barrier is maintained by multiprotein tight junction (TJ) complexes—anchored by zonula occludens-1 (ZO-1), occludin, and claudins—that regulate paracellular permeability and prevent the passage of luminal microbial content into the submucosal compartment (16). Ethanol and its primary colonic metabolite, acetaldehyde, disrupt TJ protein expression and localization through several converging mechanisms: activation of myosin light-chain kinase (MLCK), generation of reactive oxygen species (ROS), NF-κB-dependent transcriptional suppression, and phosphorylation-dephosphorylation imbalances at TJ protein binding domains (17–19).

These direct toxic effects are amplified by alcohol-induced gut dysbiosis. Chronic alcohol consumption consistently reduces the abundance of SCFA-producing taxa—including *Faecalibacterium*, *Roseburia*, *Eubacterium*, and *Lactobacillus*—while enriching LPS-bearing Gram-negative *Enterobacteriaceae* (20, 21). The net consequence is a reduction in colonic butyrate, which deprives colonocytes of their primary energy substrate, eliminates a key NF-κB-inhibitory signal in intestinal epithelial and immune cells, and impairs the transcriptional upregulation of TJ proteins, collectively amplifying barrier dysfunction (11, 22).

A clinically critical dimension of this pathology is its persistence. Longitudinal human studies have documented that ZO-1 and claudin-1 expression remain significantly reduced in the colon of abstinent individuals, with plasma LPS levels remaining elevated throughout the abstinence period (23). Studies in patients with at least 7 days of abstinence have found that colonic permeability remained elevated and that barrier resilience was reduced, even in the absence of overt liver disease (24). In a landmark study of 60 AUD patients evaluated at 2 days and 3 weeks of abstinence, intestinal permeability correlated significantly with scores for craving, anxiety, and depressive symptoms, and persistent dysbiosis at 3 weeks predicted sustained psychiatric symptom burden (14). These findings establish that leaky gut in AUD is not a reversible epiphenomenon of active drinking but a sustained biological injury that continues to drive LPS translocation—and thus peripheral and central inflammation—during the period of highest relapse risk.

It should be noted that the human evidence supporting the proposed mechanism (14, 15) derives primarily from cross-sectional and short-term longitudinal studies in individuals who already meet criteria for AUD. Therefore, although the association between increased intestinal permeability and depressive symptoms during AUD is consistent with the model proposed here, these studies cannot determine whether gut barrier dysfunction precedes depressive symptoms or whether pre-existing depressive symptoms contribute to alcohol consumption, as proposed by the self-medication hypothesis (25). Addressing this question will require prospective studies initiated before AUD onset, as well as Mendelian randomization analyses using genetic variants associated with intestinal barrier integrity or the abundance of SCFA-producing gut microbiota. Importantly, this uncertainty does not weaken the therapeutic rationale proposed in this Perspective. Regardless of the directionality of this relationship, dietary fiber may improve intestinal barrier integrity and attenuate neuroinflammatory signaling through endogenous butyrate production, supporting its use as a low-risk, mechanistically grounded adjunctive intervention.

## 4 From leaky gut to neuroinflammation: the gut-brain inflammatory cascade

LPS translocated across the compromised intestinal barrier enters the portal and systemic circulation, where it binds TLR4 on circulating monocytes and macrophages, as well as Kupffer cells in the liver, triggering the production of tumor necrosis factor-α (TNF-α), interleukin-1β (IL-1β), and IL-6 (8, 26). These cytokines access the brain parenchyma through regions of constitutively elevated blood–brain barrier (BBB) permeability—such as the circumventricular organs—and through vagal afferent signaling to limbic and hypothalamic circuits (26). Within the central nervous system (CNS), LPS-activated cytokines activate microglia and astrocytes in the prefrontal cortex (PFC), hippocampus, and nucleus accumbens (NAc), regions that govern executive function, mood regulation, and reward processing (8, 27). Importantly, ethanol itself directly activates microglial TLR4, and this central activation can persist for months after withdrawal (10), providing a neurobiological basis for the protracted psychiatric symptoms observed during abstinence in AUD.

The neurochemical consequences of this sustained neuroinflammatory state span three major neurotransmitter systems directly implicated in both MDD and AUD relapse. At the glutamatergic level, neuroinflammation suppresses astroglial glutamate transporter GLT-1 expression in the NAc and PFC, elevating extracellular glutamate, promoting excitotoxic stress, and impairing executive inhibitory control (28). At the dopaminergic level, LPS-driven microglial activation inhibits tyrosine hydroxylase (TH) activity in the ventral tegmental area. Dysbiosis-associated loss of SCFAs reduces TH expression and upregulates the dopamine transporter (DAT), collectively producing hypodopaminergic tone in the NAc that manifests clinically as anhedonia—a core feature of both depression and alcohol abstinence syndrome (29–31). At the serotonergic level, systemic inflammatory cytokines activate indoleamine 2,3-dioxygenase 1 (IDO1), diverting tryptophan away from serotonin synthesis and into the kynurenine pathway, where pro-inflammatory microglia favor conversion of kynurenine to quinolinic acid (QUIN)—a neurotoxic NMDA receptor agonist—rather than to neuroprotective kynurenic acid (KYNA) through astrocyte-mediated pathways (15, 32). In a study of 57 AUD patients undergoing detoxification, tryptophan levels correlated directly with depression severity, and SCFA-producing bacteria were positively associated with the neuroprotective KYNA/QUIN ratio (15).

Collectively, these neuroinflammatory alterations disrupt glutamatergic and dopaminergic neurotransmission while impairing synaptic plasticity, processes increasingly recognized across several neuropsychiatric disorders (33, 34). This neuroinflammatory disruption of neurotransmitter systems is also reflected in the suppression of brain-derived neurotrophic factor (BDNF), a neurotrophin essential for dendritic morphology, synaptic plasticity, and mood regulation. Proinflammatory cytokines and LPS directly suppress BDNF gene expression in the PFC and hippocampus (35). Reduced BDNF signaling via its TrkB receptor impairs CREB-dependent synaptic maintenance, contributing to dendritic atrophy and reduced synaptic density observed in both MDD- and AUD-related depression (36).

In our own animal study, 2-month-old male Sprague–Dawley rats received ethanol by intraperitoneal injection (1 g/kg/day; 30% ethanol in saline) on alternate days for 3 weeks. This route of administration was selected because of the well-documented heterogeneity of voluntary ethanol consumption in outbred Sprague–Dawley rats, in which only a subset of animals consistently self-administer high amounts of ethanol (37, 38). Ethanol exposure reduced BDNF expression in the prefrontal cortex (PFC) by approximately 33% and was accompanied by marked reductions in dendritic arborization. Both alterations were partially reversed by fenofibrate treatment after ethanol exposure (fenofibrate is a synthetic PPAR-α agonist that attenuates NF-κB signaling and neuroinflammation) (39). Extension of this work using *in vivo* microdialysis confirmed that anti-inflammatory treatment during abstinence restored dopaminergic and glutamatergic homeostasis in the NAc, directly connecting neuroinflammation control to neurotransmitter recovery (39). Collectively, these findings support the concept that recovery of neurotransmitter homeostasis and BDNF signaling may depend, at least in part, on attenuation of NF-κB-mediated neuroinflammation rather than on fenofibrate’s specific pharmacological properties. Butyrate, produced endogenously through dietary fiber fermentation, inhibits NF-κB through both histone deacetylase (HDAC)-dependent and GPR109A-mediated signaling pathways (12, 22), while simultaneously reducing gut-derived LPS, a major trigger of systemic and central inflammatory responses (11, 22). Dietary fiber, therefore, may converge on the same NF-κB-dependent neuroinflammatory pathway identified in our experimental model using this i.p. ethanol-exposed Sprague–Dawley rat paradigm (39, 40), but through an upstream physiological mechanism driven by endogenous butyrate production rather than direct pharmacological modulation (12, 41).

## 5 Dietary fiber and butyrate: restoring the gut-brain axis

### 5.1 Mechanistic rationale

Dietary fibers are non-digestible polysaccharides that reach the colon largely intact and serve as the primary fermentation substrate for resident butyrate-producing bacteria, including *Faecalibacterium prausnitzii*, *Roseburia intestinalis*, *Eubacterium rectale*, and *Butyrivibrio fibrisolvens* (22). These are precisely the taxa most consistently depleted by chronic alcohol consumption (20, 21). Fermentation of dietary fiber, particularly inulin, fructo-oligosaccharides (FOS), β-glucan, arabinoxylan, and resistant starch, yields butyrate as the principal end product, which can exert intestinal barrier-protective effects through complementary mechanisms: it serves as the primary energy substrate for colonocytes, inhibits NF-κB and HDAC in intestinal epithelial and immune cells, and transcriptionally upregulates TJ proteins, reducing paracellular permeability (12, 22).

Beyond the gut, butyrate can exert direct and indirect neuromodulatory effects relevant to AUD-associated neuroinflammation and depressive symptoms by modulating microglial activity, epigenetic regulation, BBB integrity, and neuroplasticity pathways (42). Together, these mechanisms position SCFAs as important mediators of gut-brain communication capable of attenuating neuroinflammatory signaling while supporting glial function and neurotransmitter homeostasis (43). By reducing gut-derived LPS translocation, dietary interventions may attenuate peripheral and central inflammatory signaling, thereby creating conditions that favor the preservation of astrocytic GLT-1 expression and glutamate homeostasis in the NAc and PFC (28, 33, 34). Through HDAC inhibition and cAMP-dependent mechanisms, SCFAs regulate TH expression and DAT activity, supporting dopaminergic tone in the NAc and alleviating anhedonia (30). Butyrate also inhibits IDO1 activity and shifts microglial polarization toward an anti-inflammatory phenotype, reducing QUIN production and restoring the KYNA/QUIN balance toward neuroprotection (15, 32). In enterochromaffin cells, SCFAs upregulate tryptophan hydroxylase 1 (TPH1) expression, promoting intestinal serotonin synthesis (44). Finally, dietary modulation of gut microbiota through increased fiber intake consistently elevates hippocampal and cortical BDNF expression in animal models, at least partly through HDAC inhibition by butyrate (45).

### 5.2 Preclinical evidence in alcohol models

Preclinical evidence directly demonstrating these effects in the context of alcohol exposure is compelling. Tributyrin—a butyrate prodrug with enhanced colonic bioavailability—normalized ZO-1 and occludin expression, reduced endotoxemia, and attenuated hepatic inflammation in mice subjected to chronic-binge ethanol exposure (46, 47). Butyrate supplementation also mitigated acute ethanol-induced mucosal disruption at the cellular level (48). Most relevant to drinking behavior, intragastric administration of a SCFA mixture including butyrate to genetically alcohol-preferring UChB rats reduced voluntary ethanol intake by 85–90% while simultaneously normalizing neuroinflammatory parameters in the NAc (49), suggesting that gut-brain axis restoration can directly influence the neurobiological drivers of compulsive alcohol consumption.

Complementary findings from an antibiotic-induced dysbiosis model further support the proposed gut-brain mechanism. In mice, sodium butyrate supplementation prevented the increase in ethanol consumption induced by antibiotic-mediated depletion of the gut microbiota (50). This behavioral effect was accompanied by attenuation of ethanol-and antibiotic-induced increases in hippocampal pro-inflammatory cytokines (MCP-1, TNF-α, IL-1β, and IL-6), as well as reduced microglial and astrocytic activation (51).

### 5.3 Human studies and clinical evidence

In a pilot randomized trial, inulin supplementation in AUD patients during early abstinence modulated microbiota composition—increasing *Bifidobacterium* and *Bacteroides* abundances—and improved sociability and serum BDNF levels, demonstrating the feasibility and safety of prebiotic supplementation in this population (52). Separate analyses from the same research group showed that AUD patients with greater butyrate-producing bacterial capacity during early abstinence displayed faster recovery of intestinal permeability and lower craving scores (14). Broader clinical evidence consistently demonstrates that increased dietary fiber intake elevates fecal butyrate levels, lowers systemic inflammatory markers (C-reactive protein, IL-6), and improves intestinal barrier function (53, 54). At the dietary pattern level, Mediterranean and high-fiber plant-based diets are associated with lower rates of depression and better mental health outcomes in large epidemiological cohorts (55, 56), associations consistent with the mechanistic framework proposed here, though not yet tested in AUD-specific trials.

The clinical rationale for focusing on dietary fiber rather than direct butyrate supplementation rests on pharmacokinetic and microbiological considerations. Orally administered butyrate is largely absorbed in the proximal small intestine before reaching the colon, limiting its effectiveness at the site of action. Dietary fiber, by contrast, generates butyrate *in situ* through a gradual, physiologically regulated fermentation process that supports the reestablishment of a diverse, butyrate-producing microbiota–addressing the underlying dysbiosis rather than merely supplementing one of its metabolic consequences. Additionally, increasing dietary fiber intake to recommended levels (25–38 g/day in adults) is considered safe for the general population, is inexpensive, and can be implemented through widely available foods (57).

## 6 An integrated mechanistic model and clinical implications

**Figure 1:**
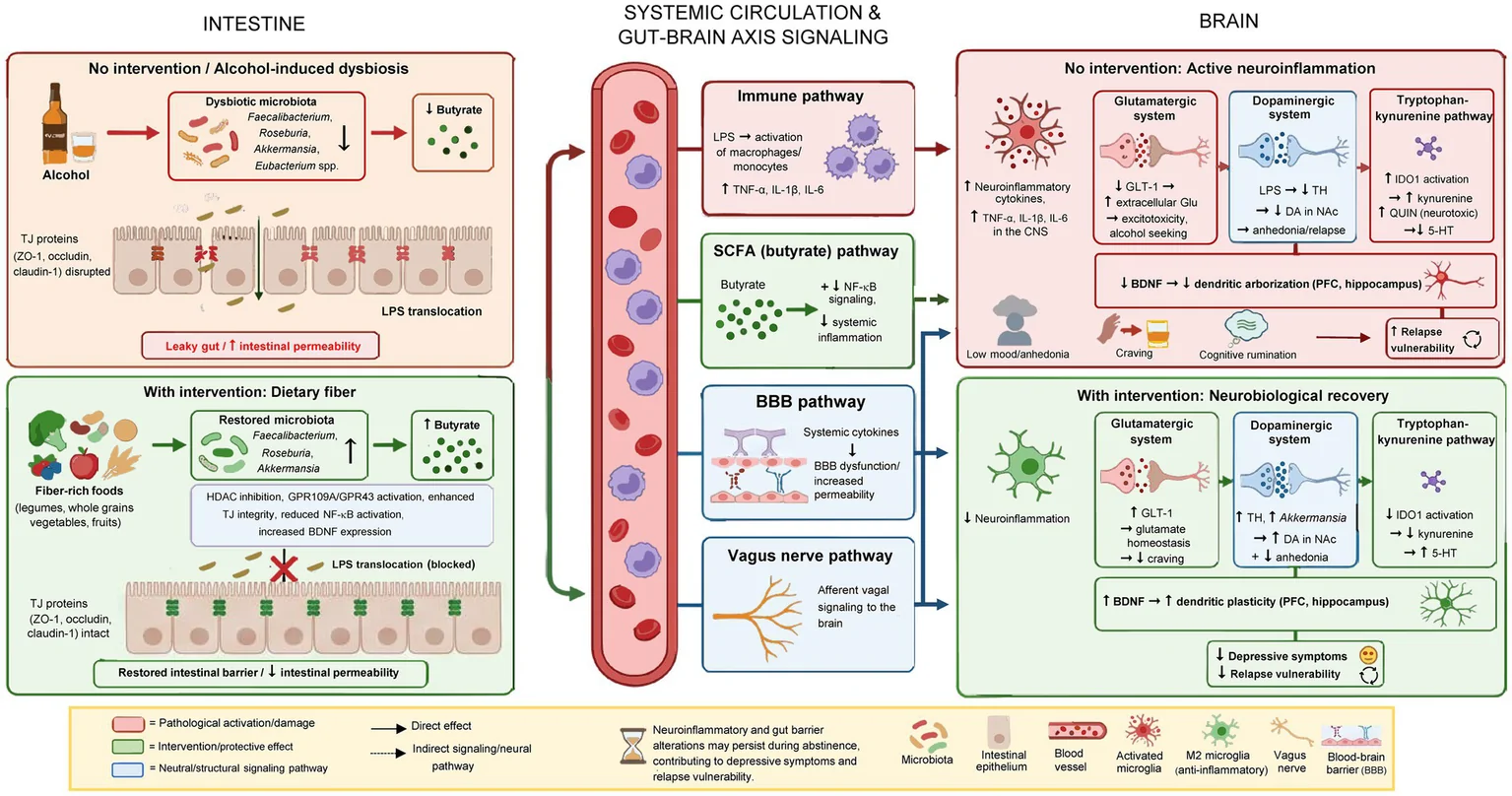
The gut-brain axis in alcohol use disorder: Mechanistic disruption and dietary fiber-mediated recovery. The figure illustrates the pathological cascade initiated by chronic alcohol consumption (upper panels, red) and its reversal by dietary fiber intervention (lower panels, green), organized across three compartments. Intestine. Alcohol-induced dysbiosis depletes butyrate-producing bacteria (*Faecalibacterium*, *Roseburia*, *Eubacterium*), reducing colonic butyrate availability and disrupting TJ proteins (ZO-1, occludin, claudin-1), thereby increasing intestinal permeability (leaky gut). Dietary fiber restores the abundance of butyrate-producing and mucus-protective taxa (including *Akkermansia*), elevating butyrate, which upregulates TJ proteins, stimulates intestinal TPH1 expression, inhibits NF-κB and HDACs, and increases BDNF via HDAC inhibition, collectively restoring barrier integrity. Systemic circulation and signaling. In the absence of intervention, translocated LPS activates macrophages and monocytes, driving systemic release of TNF-α, IL-1β, and IL-6. With dietary fiber intervention, increased circulating butyrate attenuates systemic inflammation; cytokine signaling to the brain occurs via both the blood–brain barrier and vagal afferent pathways. Brain. Neuroinflammation driven by LPS-induced cytokines produces convergent disruption of three neurotransmitter systems: (i) Glutamatergic–Suppression of GLT-1 elevates extracellular glutamate, promoting excitotoxicity and alcohol seeking; (ii) Dopaminergic–LPS inhibits TH and reduces dopamine in the NAc, producing anhedonia and relapse vulnerability; (iii) Serotonergic/kynurenine–IDO1 activation diverts tryptophan toward neurotoxic QUIN, reducing 5-HT. Reduced BDNF further impairs dendritic arborization in the PFC. Dietary fiber-mediated recovery restores GLT-1, normalizes TH and dopaminergic tone via *Akkermansia* recovery, inhibits IDO1 to redirect tryptophan toward 5-HT synthesis, elevates peripheral serotonin through enterochromaffin TPH1, and increases BDNF-dependent dendritic plasticity. The net clinical outcome shifts from depression and relapse to reduced depression and reduced relapse risk. The lateral feedback loop indicates that sustained neuroinflammation and alcohol seeking perpetuate dysbiosis in the absence of intervention, and that this circuit persists during abstinence. 5-HT, serotonin; BDNF, brain-derived neurotrophic factor; BBB, blood–brain barrier; DA, dopamine; DAT, dopamine transporter; GLT-1, glutamate transporter-1; HDAC, histone deacetylase; IDO1, indoleamine 2,3-dioxygenase 1; IL, interleukin; LPS, lipopolysaccharide; NAc, nucleus accumbens; NF-κB, nuclear factor kappa B; PFC, prefrontal cortex; QUIN, quinolinic acid; TH, tyrosine hydroxylase; TJ, tight junction; TNF-α, tumor necrosis factor-alpha; TPH1, tryptophan hydroxylase 1; ZO-1, zonula occludens-1. Figure created by the authors. This Figure was generated using FigureLabs AI and the input prompt can be found in Supplementary file 1.

Based on the evidence reviewed, the following mechanistic cascade can be articulated (Figure 1). Chronic alcohol consumption is implicated in gut dysbiosis, characterized by the depletion of SCFA-producing taxa, reduced colonic butyrate availability, and downregulation of TJ proteins. This compromises intestinal barrier integrity, increasing permeability and allowing LPS translocation into the systemic circulation, where it activates TLR4 signaling in peripheral immune cells and triggers systemic inflammation (TNF-α, IL-1β, IL-6). These circulating cytokines reach the brain through permeable regions of the BBB and via vagal afferents, activating microglia and astrocytes. This central inflammatory activation drives GLT-1 suppression, IDO1 activation, BDNF reduction, and TH/DAT dysregulation, which together produce excess extracellular glutamate, hypodopaminergic tone, serotonin deficiency, and BDNF-dependent dendritic atrophy. The cumulative result is a depressive phenotype with sustained relapse vulnerability during abstinence.

Critically, this cascade does not spontaneously resolve upon alcohol cessation. The intestinal barrier remains disrupted, LPS translocation persists, and the neuroinflammatory and neurotransmitter imbalances that sustain depression and drive relapse continue unabated. This persistence identifies a specific therapeutic window during early and extended abstinence, during which gut-targeted interventions are expected to exert their greatest benefit.

Dietary fiber intervention targets the upstream segment of this cascade: by restoring butyrate-producing microbiota, fiber rebuilds the colonocyte energy supply, upregulates TJ proteins, reduces paracellular LPS flux, and inhibits NF-κB at the intestinal immune level—preventing the initiation of the peripheral-to-central inflammatory signal. This upstream positioning complements rather than replaces pharmacological interventions targeting downstream steps, such as anti-inflammatory agents (e.g., PPAR-α agonists), glutamate modulators, or antidepressants. For patients with AUD-related depression who respond inadequately to antidepressant monotherapy, dietary fiber represents a safe, additive therapeutic lever that addresses a root mechanism of both conditions simultaneously.

In practice, dietary fiber intake should be systematically assessed and promoted from the outset of the detoxification phase. Fiber-rich foods include legumes (lentils, chickpeas, beans), whole grains (oats, barley), inulin-rich vegetables (artichoke, onion, garlic, asparagus), and fermentable fruits (apples, berries, bananas). For patients unable to achieve adequate intake through dietary modification, prebiotic supplementation with inulin, FOS, or β-glucan represents an accessible and well-tolerated alternative with demonstrated safety in AUD populations (52).

## 7 Discussion

AUD-related depression and relapse vulnerability share a neuroinflammatory substrate driven, in substantial part, by alcohol-induced disruption of the gut-brain axis. The depletion of butyrate-producing bacteria, reduction in intestinal SCFA availability, TJ protein downregulation, and LPS-driven central neuroinflammation collectively produce the glutamatergic, dopaminergic, and serotonergic imbalances—and the BDNF deficits—that underlie the depressive phenotype and compulsive alcohol seeking during abstinence. Dietary fiber intake, by promoting the growth of butyrate-producing microbiota *in situ*, targets this pathological cascade at its intestinal origin: strengthening the epithelial barrier, reducing endotoxemia, inhibiting NF-κB-driven neuroinflammation, and supporting the recovery of neurotransmitter homeostasis and BDNF-dependent synaptic plasticity.

Based on the evidence reviewed, we propose that intestinal barrier dysfunction represents an actionable upstream therapeutic target linking gut dysbiosis, neuroinflammation, depression, and relapse vulnerability in AUD. The mechanistic coherence of this proposal is supported by a convergent body of preclinical and human data, though randomized clinical trials specifically designed to evaluate the effect of fiber-rich dietary interventions on depression scores, craving, and relapse rates in AUD populations remain an unmet need. Future research priorities include: (i) prospective trials with prebiotic fiber supplementation in AUD patients during early abstinence, using intestinal permeability markers (LPS-binding protein, zonulin), kynurenine pathway metabolite ratios (KYNA/QUIN), and inflammatory cytokine profiles as mechanistic endpoints alongside clinical outcomes; (ii) microbiome profiling to identify which fiber subtypes and fermentation products are most effective in reconstituting butyrate-producing taxa depleted by alcohol; and (iii) assessment of whether fiber-mediated gut-brain axis restoration potentiates the efficacy of pharmacological anti-inflammatory strategies in reducing AUD-related depression and relapse. These studies have the potential to transform a mechanistically grounded nutritional hypothesis into an evidence-based clinical recommendation for the management of one of the most prevalent and refractory comorbidities in addiction medicine. Instead of targeting downstream neuropsychiatric consequences of alcohol use disorder, gut-directed nutritional interventions may target an upstream driver, intestinal barrier dysfunction, to mitigate neuroinflammation, depression, and relapse vulnerability.

## Data Availability

The original contributions presented in the study are included in the article/Supplementary material, further inquiries can be directed to the corresponding authors.
