# Supplementary material for: The gut-brain axis as a therapeutic target in alcohol use disorder: dietary fiber, intestinal barrier integrity, and neuroinflammatory mechanisms underlying depression
Source: Front Nutr. 2026 Sep 1;13:1927800. doi: 10.3389/fnut.2026.1927800 (PMC13574582; doi:10.3389/fnut.2026.1927800)

Supplementary material: Generative AI input prompt and output

Manuscript figure: Figure 1 - "The gut-brain axis as a therapeutic target in alcohol use disorder: dietary fiber, intestinal barrier integrity, and neuroinflammation-driven depression"

AI-based platform: FigureLabs (https://chat.figurelabs.ai)

Prompt authorship: The prompt was authored by the corresponding author.

Note: The output shown below reflects the raw AI-generated asset prior to minor manual formatting adjustments made by the author for the final published version.

1. Input prompt

Create a publication-ready biomedical schematic (wide landscape, white background) titled "The gut-brain axis as a therapeutic target in alcohol use disorder: dietary fiber, intestinal barrier integrity, and neuroinflammation-driven depression."

3 columns: INTESTINE | SYSTEMIC CIRCULATION & SIGNALING | BRAIN. Columns 1 and 3 split top (red, "No intervention") / bottom (green, "With intervention").

COLUMN 1: Red—alcohol → dysbiosis (↓*Faecalibacterium*, *Roseburia*, *Akkermansia*) → ↓Butyrate → disrupted tight junctions (ZO-1, occludin, claudin-1) → LPS leak → "Leaky gut". Green—fiber foods → restored microbiota → ↑Butyrate → intact tight junctions, LPS blocked → "Restored barrier". Red feedback arrow on left looping bottom to top.

COLUMN 2: Vertical blood vessel with 4 boxes: red "Immune pathway" (LPS→macrophages→↑TNF-α/IL-1β/IL-6); green "SCFA pathway" (Butyrate→↓NF-κB); blue "BBB pathway" (cytokines→permeability); blue "Vagus nerve pathway" (afferent signaling).

COLUMN 3: Red—activated microglia; 3 boxes: Glutamatergic (↓GLT-1→excitotoxicity), Dopaminergic (↓TH/DA→anhedonia), Tryptophan-kynurenine (↑IDO1/QUIN→↓5-HT); ↓BDNF→↓dendrites; flow: Neuroinflammation→Neurotransmitter imbalance→Depression→Relapse. Green—M2 microglia; same 3 boxes reversed (↑GLT-1, ↑TH/DA, ↓IDO1/↑5-HT); ↑BDNF→↑dendrites; flow ending ↓Depression/↓Relapse.

Bottom legend: colors (red=pathological, green=protective, blue=neutral pathway), arrows (solid=direct, dashed=indirect), abbreviations (NAc, Glu, IDO1, QUIN, BDNF).

Style: high resolution, clean BioRender-style biomedical icons, bold sans-serif, sharp legible text.

2. Generated output (raw AI output, prior to author's manual adjustments)

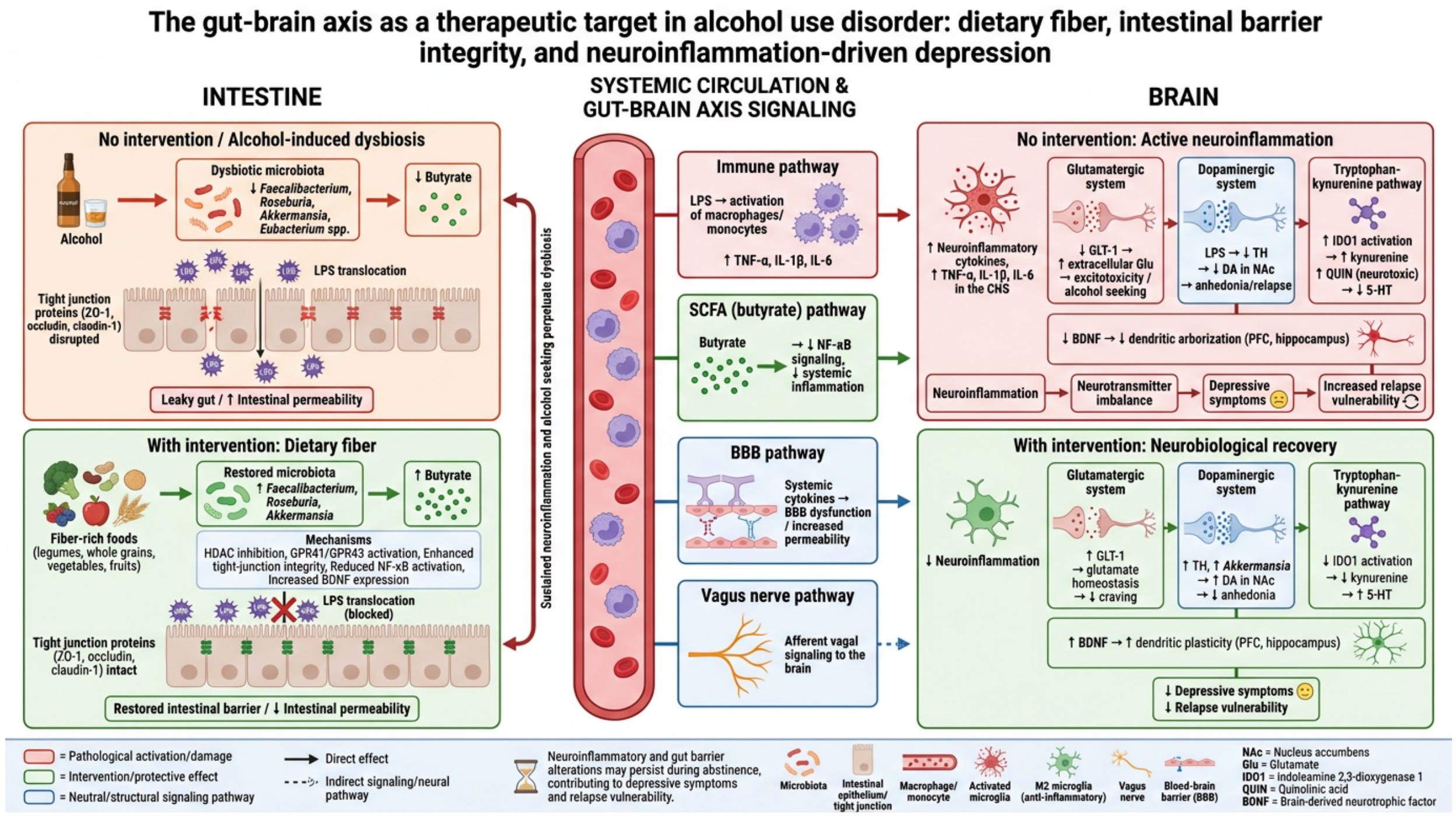

Supplement: Supplementary file 1 [file Supplementary_file_1.pdf]
